# A Machine Learning-Based Radiomics Model for the Differential Diagnosis of Benign and Malignant Thyroid Nodules in F-18 FDG PET/CT: External Validation in the Different Scanner

**DOI:** 10.3390/cancers17020331

**Published:** 2025-01-20

**Authors:** Junchae Lee, Jinny Lee, Bong-Il Song

**Affiliations:** 1 Keimyung University School of Medicine, Daegu 42601, Republic of Korea; 5596150@stu.kmu.ac.kr; 2 Department of Nuclear Medicine, Keimyung University Dongsan Hospital, Daegu 42601, Republic of Korea; jinnylee3282@gmail.com; 3 Department of Medical Information, Keimyung University School of Medicine, Daegu 42601, Republic of Korea

**Keywords:** thyroid incidentalomas, radiomics, feature selection, prediction, F-18 FDG PET/CT

## Abstract

Background/Objectives: Accurate diagnosis is essential to avoid unnecessary procedures for thyroid incidentalomas (TIs). Advances in radiomics and machine learning applied to medical imaging offer promise for assessing thyroid nodules. This study utilized radiomics analysis on F-18 FDG PET/CT to improve preoperative differential diagnosis of TIs. Methods: A total of 152 patient cases were retrospectively analyzed and split into training and validation sets (7:3) using stratification and randomization. Results: The least absolute shrinkage and selection operator (LASSO) algorithm identified nine radiomics features from 960 candidates to construct a radiomics signature predictive of malignancy. Performance of the radiomics score was evaluated using receiver operating characteristic (ROC) analysis and area under the curve (AUC). In the training set, the radiomics score achieved an AUC of 0.794 (95% CI: 0.703–0.885, *p* < 0.001). Validation was performed on internal and external datasets, yielding AUCs of 0.702 (95% CI: 0.547–0.858, *p* = 0.011) and 0.668 (95% CI: 0.500–0.838, *p* = 0.043), respectively. Conclusions: These results demonstrate that the selected nine radiomics features effectively differentiate malignant thyroid nodules. Overall, the radiomics model shows potential as a valuable predictive tool for thyroid cancer in patients with TIs, supporting improved preoperative decision-making.

## Simple Summary

The integration of advanced imaging techniques and radiomics analysis represents a promising direction in thyroid nodule management. Radiomics plays a pivotal role in differentiating between cancerous and benign lesions by offering a deeper, more nuanced analysis of medical images. By quantifying tumor heterogeneity and providing objective, standardized metrics, radiomics captures subtle tissue characteristics that may elude visual inspection. This study aimed to improve the preoperative differentiation of thyroid incidentalomas (TIs) using radiomics analysis on F-18 FDG-PET/CT. Of 960 radiomics features, nine key features were selected using the LASSO algorithm to create a radiomics score. The score demonstrated good predictive performance for identifying malignant thyroid nodules. This model shows promise for aiding in the diagnosis of thyroid cancer.

## 1. Introduction

Thyroid nodules are a prevalent clinical issue, with their incidence rising globally [1]. These nodules, often detected incidentally during imaging for non-thyroidal conditions, are termed thyroid incidentalomas (TIs) [2]. F-18 fluorodeoxyglucose (FDG) positron emission tomography (PET) has established itself as a valuable functional imaging technique. It offers exceptional capabilities in detecting primary cancers [3,4], guiding treatment planning and monitoring [5,6,7], predicting prognosis [8,9,10,11], identifying early recurrence [12], and diagnosing regional lymph node involvement and distant metastases [13,14,15,16]. F-18 FDG PET/CT imaging has identified focal TIs in 1–4% of both cancer patients and healthy individuals, with associated cancer risks ranging from 14–50% [17,18,19,20]. While ultrasonography (US) and fine-needle aspiration (FNA) biopsy can determine that most nodules are benign [21], accurate characterization remains challenging. Definitive diagnosis frequently necessitates surgical intervention, which can lead to scarring and impair normal thyroid function [22]. Consequently, precise preoperative evaluation is essential to accurately select patients who require biopsy or surgery, thus reducing unnecessary procedures and related complications.

Many studies have suggested that PET-derived conventional parameters, such as standardized uptake value, metabolic tumor volume, and total lesion glycolysis, are helpful in differentiating benign and malignant TIs [23,24,25]. These parameters may increase diagnostic accuracy and decrease the need for invasive diagnostic procedures such as FNA or surgery. However, it is important to acknowledge that solely relying on PET-derived conventional parameters for differentiating malignant thyroid tumors is still challenging, and further research is necessary to enhance the accuracy of the diagnosis.

Radiomics is a rapidly developing field of study that aims to extract quantitative data from medical images, such as PET and CT [26]. To access radiomic information that cannot be seen in standard medical images, advanced texture and shape analysis techniques are required. Texture analysis refers to diverse mathematical models used to assess the relationships between the signal intensity of pixels and their relative position in the image [27]. By analyzing radiomics features extracted from medical images, novel diagnostic and prognostic markers that can aid in the management of TIs could be identified [28]. Thus, radiomics features extracted from F-18 FDG-PET/CT hold great promise in the diagnosis and management of thyroid nodules. Radiomics and machine learning techniques applied to medical imaging have the potential to improve diagnostic accuracy, reduce unnecessary invasive procedures, and ultimately improve patient outcomes.

Therefore, this study aims to develop and validate a machine learning-based radiomics model for distinguishing malignant from benign thyroid nodules using F-18 FDG PET/CT images.

## 2. Materials and Methods

### 2.1. Patients

**Figure 1:**
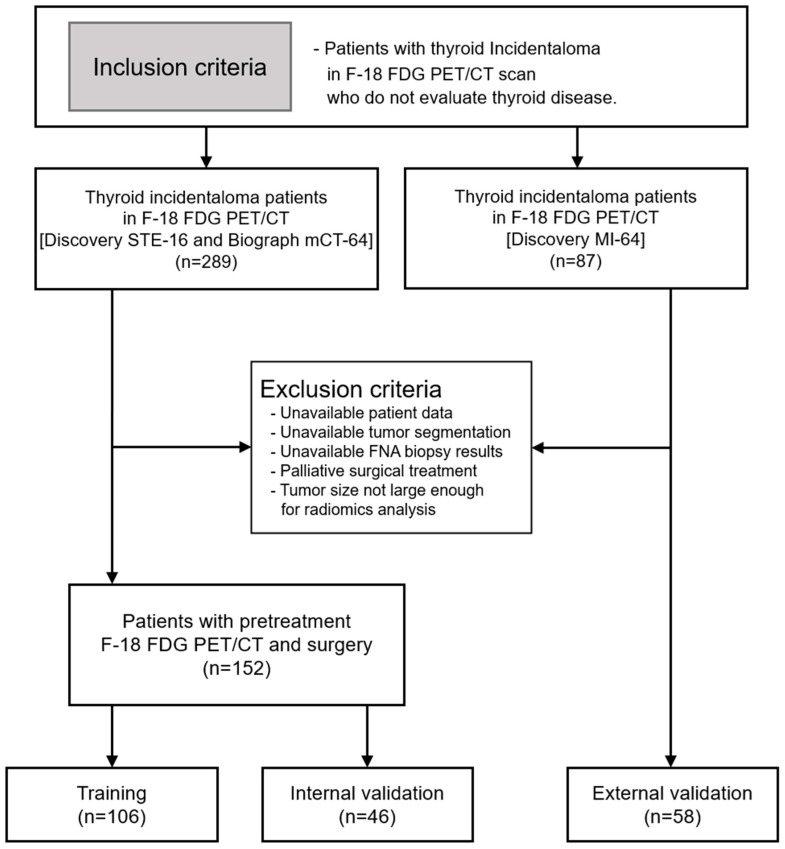
Flow diagram of patient selection. Of the 376 patients who have thyroid incidentaloma in the F-18 FDG PET/CT, the final cohort was divided into a training set (n = 106), an internal validation set (n = 46), and an external validation set (n = 58) for model development and validation.

We retrospectively collected data from 289 consecutive patients with TIs who underwent F-18 FDG PET/CT between January 2010 and August 2014 at Keimyung University Dongsan Hospital. Patients who underwent an F-18 FDG PET/CT exam for staging or restaging purposes of various diseases, excluding those aimed at evaluating thyroid tumors, and who had performed FNA were enrolled in this study. The exclusion criteria were as follows: (1) unavailable patient data; (2) unavailable tumor segmentation; (3) unavailable FNA biopsy results; and (4) tumor size not large enough for radiomics analysis. A total of 152 patient cases were eligible in this study, and they were stratified and randomly divided into 7:3 training and internal validation sets. Both training and internal validation sets contained approximately the same malignant and benign ratio. An additional 58 patient cases obtained using different PET/CT scanners between April 2019 and April 2022 were used as an external validation set (Figure 1). All patient data were anonymized before analysis. FNA results were classified according to Bethesda categories, and histopathological outcomes post-thyroidectomy were recorded to confirm the final diagnosis. FNA-positive cases included Bethesda categories V and VI, while FNA-negative cases included categories I, II, III, and IV. The final diagnosis for malignant thyroid nodules was confirmed through histopathological examination after thyroidectomy in all patient cohorts, including the external validation group. Benign thyroid nodules were diagnosed based on FNA results for cases classified as Bethesda I–IV, with thyroidectomy performed in select cases. The present retrospective study was approved by the Institutional Review Boards of Keimyung University Dongsan Hospital, and the need for informed consent was waived.

### 2.2. F-18 FDG PET/CT Image Acquisition and Radiomics Feature Extraction

All patients underwent F-18 FDG PET/CT following a minimum 6 h fasting period and with blood glucose levels below 150 mg/dL. The images collected using the Discovery STE-16 (GE Healthcare, Milwaukee, WI, USA) and the Biograph mCT-64 (Siemens Healthcare, Knoxville, TN, USA) were used as a training and internal validation set. And the images obtained using the Discovery MI-64 (GE Healthcare, Milwaukee, WI, USA) were used as an external validation set.

The Discovery STE-16 PET/CT scanner captured images with a slice thickness of 3.75 mm over a longitudinal field of view (FOV) of 780 mm and a transaxial FOV of 700 mm, with a matrix size of 128 × 128. The spatial resolution in air was 4.29 mm full-width half-maximum (FWHM). The Biograph mCT-64 PET/CT scanner acquired images with a 3 mm slice thickness over a longitudinal FOV of 780 mm and a transaxial FOV of 700 mm, with a matrix size of 256 × 256. The spatial resolution in the air was 4 mm FWHM. The Discovery MI-64 PET/CT scanner got images with a slice thickness of 2.79 mm over a FOV of 700 mm and a transaxial FOV of 700 mm, with a matrix size of 256 × 256. The spatial resolution in air was 2.1 mm FWHM. All PET images were resampled to an isotropic voxel of 2 × 2 × 2 cubic millimeters using a thresholding 3D segmentation-based method for imaging standardization prior to radiomics feature extraction.

F-18 FDG PET images were carefully analyzed by a well-experienced nuclear medicine physician. Thyroid incidentaloma, defined by increased focal thyroid uptake compared to surrounding tissues, was segmented by drawing a volume of interest (VOI) with a fixed threshold of 2.5 of SUV to enhance inter-observer reproducibility. Image segmentation was performed using the 3D Slicer software (Harvard Medical School, version 5.2.1). Radiomics features were extracted from each segmented thyroid incidentaloma using the PyRadiomics package implemented in Python (version 3.0.1) [29]. Each set was normalized using the min–max normalization method.

### 2.3. Radiomics Feature Selection and Radiomics Score Calculation

The least absolute shrinkage and selection operator (LASSO) was utilized to select the most useful predictive features from the 960 extracted radiomics features of PET/CT images of thyroid incidentaloma patients. The radiomics feature selection process involved three key steps: initial extraction of 960 features, normalization using min–max scaling, and feature reduction via LASSO logistic regression with 10-fold cross-validation to identify the most predictive features. Using the training data, the LASSO model was trained and cross-validated using least-squares penalty, α value of 1 and identified optimal cross-validated lambda value to determine the most useful features for constructing a radiomics score for each image. A radiomics score was calculated for each patient using a linear combination of selected features weighted by their respective LASSO coefficients to assess the likelihood of malignancy of thyroid incidentaloma. The predictive accuracy of the radiomics score was evaluated in the internal and external validation sets by the receiver operating characteristic (ROC) curve (AUC). The radiomics score formula was consistent for all selected features, as it represents a linear combination of feature values weighted by their respective LASSO coefficients. No feature was excluded or differently treated post-selection.

### 2.4. Statistical Analysis

Numeric data are expressed as the mean ± standard deviation. C-statistics were used to compare the AUC effectively. LASSO logistic regression was chosen for feature selection due to its strengths in handling high-dimensional datasets, such as those in radiomics, by applying an L1 regularization penalty. This approach effectively eliminates less relevant features, thereby reducing overfitting and enhancing model interpretability. All statistical analyses were performed using the R software (version 4.1.3, https://www.r-project.org, accessed on 10 March 2022). A *p* < 0.05 was considered statistically significant.

## 3. Results

### 3.1. Patient Characteristics

A total of 152 patients were analyzed as the training set and internal validation set, split in a 7:3 ratio, and 58 patients were used as the external validation set. The proportion of malignant cases was 41 of 106 (38.7%) in the training set, 18 of 46 (39.1%) in the internal validation set, and 11 of 58 (20%) in the external validation set. During the study period, a total of 27,319 PET/CT scans were performed, among which TIs were identified in 289 cases (prevalence: 1.1%). The clinical characteristics of enrolled patients in the training, internal validation, and external validation set are presented in Table 1. Table 2 shows the relationship between FNA/US findings and histological outcomes. Of the malignant cases, histological analysis revealed the following tumor types of post-thyroidectomy: 59 cases of papillary thyroid carcinoma, 9 cases of follicular thyroid carcinoma, and 2 cases of other malignancies. Among benign cases, 17 cases of follicular adenoma and 12 cases of nodular hyperplasia.

### 3.2. Radiomics Feature Selection

**Figure 2:**
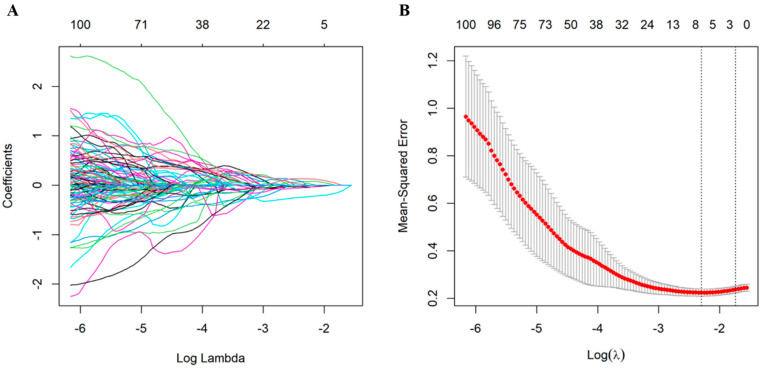
LASSO (least absolute shrinkage and selection operator) regression analysis used for feature selection. (**A**) For 980 radiomics features, the LASSO coefficient profiles are shown. The coefficient profiles of the variables as a function of the regularization parameter (Log Lambda). (**B**) The mean-squared error (MSE) versus Log Lambda, with red dots representing the MSE for each value of Lambda and error bars indicating the standard deviation. The two vertical dashed lines mark the optimal Lambda values: the left line corresponds to the minimum MSE; while the right line represents the largest Lambda within one standard error of the minimum MSE.

A total of 960 radiomics features were extracted from each VOI of the tumor on PET/CT images, and 9 features with non-zero coefficients were selected based on the LASSO logistic (Table 3). The radiomics score of each patient was calculated with selected radiomics features and respective LASSO coefficients (Figure 2). The example formula for calculating radiomics score was as follows:

Radiomics score = Intercept + c1 × feature1 + c2 × feature2 + … + c9 × feature9

Here, c1, c2, …, and c9 represent the coefficients assigned to each selected radiomics feature, and feature1, feature2, …, and feature9 are the respective values of selected features. The constructed LASSO model has an optimal range of parameter values for minimizing the mean squared error.

### 3.3. Radiomics Score Performance Evaluation

**Figure 3:**
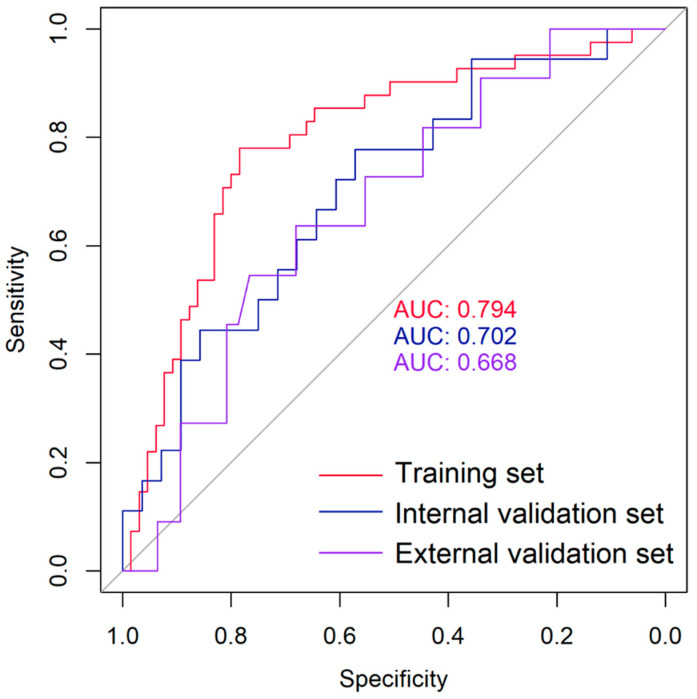
Receiver operating characteristic (ROC) curve of the radiomics score. The radiomics score showed good performance in the training set with an area under the curve (AUC) of 0.794, an AUC of 0.702 in the internal validation set, and an AUC of 0.668 in the external validation set.

The radiomics score showed good performance in the training set with an AUC of 0.794 (95% confidence interval (CI): 0.703–0.885, *p* < 0.001). The optimal cutoff value for the radiomics score was determined to be 0.40, sensitivity was 0.7846, specificity was 0.7805, positive predictive value (PPV) was 0.8500, and negative predictive value (NPV) was 0.6957, which was validated in the internal and external validation set with, respectively, an AUC of 0.702 (95% CI: 0.547–0.858, *p* = 0.011), a sensitivity of 0.5714, a specificity of 0.777, a PPV of 0.8000, and an NPV of 0.5385; and an AUC of 0.668 (95% CI: 0.500–0.838, *p* = 0.043), a sensitivity of 0.6809, a specificity of 0.6364, a PPV of 0.8889, and an NPV of 0.3182 (Figure 3).

## 4. Discussion

In the present study, the radiomics score was calculated via a formula, including nine radiomics features selected by LASSO logistic. It demonstrates strong predictive accuracy in the training set, with an AUC of 0.794 (95% confidence interval (CI): 0.703–0.885, *p* < 0.001). It was also statistically significant in the internal validation set, with an AUC of 0.702 (95% CI: 0.547–0.858, *p* = 0.011), and in external validation set, with an AUC of 0.668 (95% CI: 0.500–0.838, *p* = 0.043). The results indicate that the developed radiomics model, based on nine selected features, can effectively distinguish between benign and malignant thyroid nodules on F-18 FDG PET images. In this study, the decision to perform thyroidectomy was primarily based on FNA and Thyroid Imaging Reporting and Data System (Ti-RADS) findings. Malignant nodules classified as FNA-positive (Bethesda V–VI) were confirmed histologically after surgery. For FNA-negative nodules (Bethesda I–IV), thyroidectomy was performed in select cases. This approach introduces an inherent selection bias, as most benign nodules did not undergo surgical confirmation. Consequently, the sensitivity of FNA appears artificially high in this study since the absence of histological validation for many benign cases limits the ability to assess false negatives. Future studies are necessary to better evaluate the true diagnostic performance of FNA and its integration with radiomics and Ti-RADS classifications.

To differentiate between benign and malignant thyroid incidentaloma, invasive procedures such as FNA or surgical biopsy have been essential. To minimize invasive procedures, a need for differentiation between malignant and benign lesions in imaging findings has been suggested. Furthermore, an accurate diagnosis of cytologically indeterminate thyroid nodules is crucial to ensure the timely diagnosis of malignant or borderline tumors. A review study including eight eligible studies showed that the mean SUVmax for the 73 benign lesions was 4.6 ± 2.1, and for the 52 malignant lesions, the mean SUVmax was 6.8 ± 4.6 (*p* < 0.001) [30]. On the other hand, several studies showed no significant difference in SUVs between benign and malignant thyroid lesions [31]. The results of present study showed that SUVmean was higher in malignant thyroid lesions compared to benign ones in the training set, but neither SUVmax nor SUVmean was statistically significant in discriminating between benign and malignant thyroid lesions in TIs.

Radiomics is a rapidly developing technology that extracts complex, multi-dimensional features from clinical images [9,14,29]. It provides insights into the original shape, spectral characteristics, grayscale patterns, and inter-pixel relationships within these images. Several radiomics studies have successfully predicted malignancy in thyroid nodules using F-18 FDG PET imaging. Ko et al. report that F-18 FDG PET/CT-based radiomics features showed good diagnostic performance in predicting malignant thyroid nodules [32]. Their study found a pooled sensitivity of 0.77 and specificity of 0.67, with a positive likelihood ratio of 2.3 and negative likelihood ratio of 0.35 in the meta-analysis. These findings demonstrate the potential of radiomics in distinguishing between malignant and benign thyroid nodules in F-18 FDG PET imaging. However, given the clinical situation with different PET scanners and imaging acquisition protocols, it is essential to have external validation that can be applied in different environments. Dondi et al. also demonstrate the importance of selection of good radiomics features for the prediction of final nature of TIs on F-18 FDG PET images [33]. In this study, we suggest a new radiomics score, which was statistically significant in the training set, the internal validation set, and the external validation set. The external validation dataset contains data from a different PET scanner from that used for the training set and the internal validation set. The variability in feature distributions between scanners highlights the need for more robust normalization techniques. Future studies should focus on harmonizing imaging protocols or developing scanner-invariant feature extraction methods.

In predicting malignant versus benign lesions using F-18 FDG PET images, machine learning models that integrate radiomics features demonstrate significant advantages over traditional single predictors such as SUVmax [34]. Radiomics enables the extraction of a wide array of image characteristics, including texture, shape, and intensity patterns, offering a more nuanced and comprehensive evaluation of tumor heterogeneity and behavior [35]. This multidimensional analysis leads to enhanced predictive accuracy and model robustness. However, radiomics also has limitations, including the potential for overfitting due to the high dimensionality of data, the need for large and diverse datasets to validate models, and variability in feature extraction methods, which can impact the reproducibility and generalizability of the findings across different institutions and imaging protocols. In the present study, the AUCs for the training and internal validation sets were good, but the results for the external validation set were relatively poor, albeit statistically significant. This may be due to the low incidence of malignant patients in the external validation set, but it may also be due to the limitations of radiomics features.

**Figure 4:**
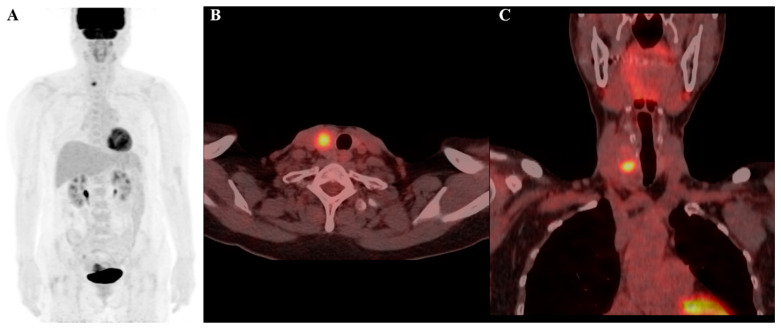
F-18 FDG PET/CT image of a 69-year-old female patient, highlighting a thyroid incidentaloma in the right lobe of the thyroid gland: (**A**) maximum intensity projection (MIP); (**B**) axial PET/CT image; and (**C**) coronal PET/CT image. The lesion demonstrates increased FDG uptake with a maximum standardized uptake value (SUVmax) of 9.2. Radiomic features include a log-sigma-2-0-mm-3D_glszm_SmallAreaEmphasis value of 0.553 (relatively low) and a wavelet-HLH_gldm_LargeDependenceLowGrayLevelEmphasis value of 33.814 (relatively high). Despite the high SUVmax, the final diagnosis confirmed the nodule to be benign.

**Figure 5:**
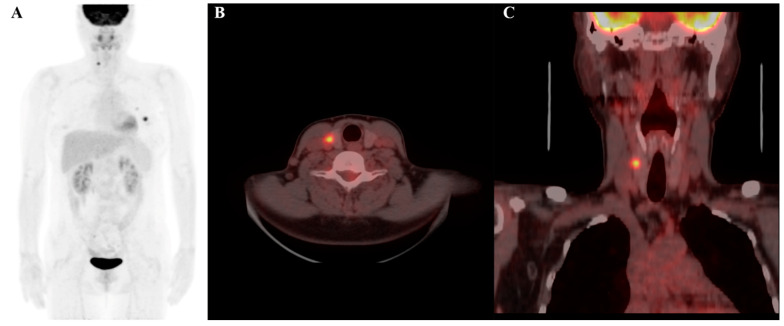
F-18 FDG PET/CT image of a 56-year-old female patient, showing a thyroid incidentaloma located in the right lobe of the thyroid gland: (**A**) maximum intensity projection (MIP); (**B**) axial PET/CT image; and (**C**) coronal PET/CT image. The lesion exhibits elevated FDG uptake, with a maximum standardized uptake value (SUVmax) of 7.3. Radiomic analysis reveals a log-sigma-2-0-mm-3D_glszm_SmallAreaEmphasis value of 0.734 (relatively high) and a wavelet-HLH_gldm_LargeDependenceLowGrayLevelEmphasis value of 4.440 (relatively low). The final diagnosis confirmed that the nodule was malignant.

The listed radiomics features are valuable for cancer diagnosis as they capture specific tumor texture and intensity patterns associated with malignancy. These features help assess tumor heterogeneity, an indicator of aggressive behavior. In particular, the large-dependence low-gray-level emphasis (LDLGLE) in the gray level-dependence matrix (GLDM) feature is related to areas where low-intensity signals (often seen in less metabolically active regions) depend on surrounding pixel values, which can be indicative of the structural characteristics of a tumor [36]. Benign tumors are often more homogeneous and may have lower gray-level textures, which might increase the value of features like LDLGLE, whereas malignant tumors are generally more heterogeneous, with more irregular and higher intensity patterns, potentially leading to lower values for this feature. Features such as LDLGLE are indicative of tumor heterogeneity, reflecting areas where low-intensity signals depend on surrounding pixel values. In the context of thyroid nodules, this feature may correspond to regions with less metabolically active tissue that are structurally heterogeneous—an attribute often associated with malignant lesions. These characteristics align with the known biological behavior of thyroid cancers, which typically exhibit greater textural and metabolic irregularity compared to benign nodules. Thus, this feature provides insight into tumor composition and behavior (Figure 4 and Figure 5).

The small size of the external validation set, and the low proportion of malignancies (20%) likely contributed to the reduced AUC. Future studies should include larger and more balanced datasets to enhance the generalizability of the model. Oversampling techniques, such as the synthetic minority over-sampling technique (SMOTE), could address the class imbalance observed in our dataset by generating synthetic samples of underrepresented classes. Preliminary studies have demonstrated the potential of SMOTE to enhance model performance, particularly in imbalanced datasets. Future research should evaluate the impact of such techniques on the diagnostic accuracy of radiomics-based models for thyroid nodules, particularly in external validation cohorts where malignancy prevalence is low.

There are some limitations in this study. First, all 152 patient cases in the study are from a single center. Also, patients who underwent FNA were included in our study. These may cause a potential selection bias. The model’s performance in both internal and external validation sets highlight its potential for clinical application. However, further prospective studies with larger cohorts are necessary to confirm these findings and refine the model for broader clinical use. Second, there is a possibility that other machine learning methods will produce better results. We selected nine radiomics features using LASSO logistic regression. Machine learning methods other than LASSO logistic regression need to be validated. Lastly, the sample size may not be sufficient. To validate our findings, further studies with larger external cohorts are necessary.

## 5. Conclusions

This study successfully developed and validated a machine learning-based radiomics model for the differential diagnosis of thyroid nodules. The model demonstrated good predictive accuracy and robustness, suggesting its potential utility in clinical settings to improve the management of patients with TIs.

## Figures and Tables

**Table 1:** Patient characteristics in the training and validation sets.

Characteristics	Training Set (*n* = 106)			Internal Validation Set (*n* = 46)			External Validation Set (*n* = 58)		
	Benign (*n* = 65)	Malignant (*n* = 41)	*p*	Benign (*n* = 28)	Malignant (*n* = 18)	*p*	Benign (*n* = 47)	Malignant (*n* = 11)	*p*
Age (years)	63.0 ± 11.4	61.5 ± 10.5	0.484	65.9 ± 9.2	60.3 ± 13.1	0.097	61.8 ± 12.9	64.9 ± 9.2	0.460
Sex			1.000			0.694			1.000
Female	45 (69.2%)	28 (68.3%)		19 (67.9%)	14 (77.8%)		37 (78.7%)	9 (81.8%)	
Male	20 (30.8%)	13 (31.7%)		9 (32.1%)	4 (22.2%)		10 (21.3%)	2 (18.2%)	
Size (mm)	18.8 ± 11.9	17.7 ± 11.6	0.639	18.8 ± 10.0	18.7 ± 12.6	0.982	20.9 ± 12.1	25.6 ± 31.7	0.635
SUVmax	7.5 ± 7.3	10.0 ± 7.7	0.103	7.9 ± 6.7	8.2 ± 5.9	0.888	9.3 ± 5.5	11.1 ± 8.5	0.524
SUVmean	3.5 ± 1.2	4.1 ± 1.4	0.034	3.6 ± 0.9	3.7 ± 1.1	0.646	4.1 ± 1.2	4.3 ± 1.0	0.686
MTV (mm^3^)	482.4 ± 183.8	319.9 ± 85.2	<0.001	466.3 ± 167.9	306.4 ± 94.8	<0.001	503.5 ± 187.1	299.3 ± 110.9	0.458
TLG	11,116.5 ± 24,145.5	13,784.6 ± 27,457.1	0.601	11,210.0 ± 19,462.6	6348.8 ± 7099.5	0.237	13,781.2 ± 19,780.2	51,231.9 ± 149,442.5	0.426

The data are presented as mean ± standard deviation. MTV, metabolic tumor volume; SUV, standardized uptake value; TLG, total lesion glycolysis.

**Table 2:** Distribution of FNA classes and Ti-RADS scores in the training, internal validation, and external validation sets.

Study Set	Total Cases	FNA Positive (Bethesda V and VI)	FNA Negative (Bethesda I–IV)	Ti-RADS ≥ 4	Ti-RADS < 4	Malignant Cases (Histology)
Training Set	106	44	62	94	12	41
Internal Validation	46	19	27	40	6	18
External Validation	58	12	46	48	10	11

FNA, fine-needle aspiration; Ti-RADS, Thyroid Imaging Reporting and Data System.

**Table 3:** Selected radiomics features from LASSO logistic regression analysis.

Radiomics Features	Coefficients
Intercept	0.338331853
log-sigma-2-0-mm-3D_glszm_SmallAreaEmphasis	0.077752305
log-sigma-3-0-mm-3D_glrlm_RunLengthNonUniformityNormalized	0.04486966
log-sigma-3-0-mm-3D_glrlm_ShortRunEmphasis	0.002361339
wavelet-LHH_glrlm_LongRunLowGrayLevelEmphasis	−0.013308531
wavelet-LHH_glszm_GrayLevelNonUniformityNormalized	−0.035576602
wavelet-LHH_glszm_SmallAreaEmphasis	0.00323665
wavelet-HLH_glrlm_ShortRunEmphasis	0.129276943
wavelet-HLH_gldm_LargeDependenceLowGrayLevelEmphasis	−0.235840268
wavelet-HHL_gldm_LargeDependenceLowGrayLevelEmphasis	−0.048990086

## Data Availability

Data supporting the present study are available from the corresponding author upon reasonable request.
